# Homœopathy or Eclecticism?

**Published:** 1882-08

**Authors:** 


### HOMŒOPATHIC PHYSICIAN.

A MONTHLY JOURNAL OF MEDICAL SCIENCE.

"If our school ever gives up the strict inductive method of Hahnemann, we are lost, and deserve only to be mentioned as a caricature, in the history of medicine."—CONSTANTINE HERING.

Vol. II. AUGUST, 1882. No. 8.

### EDITORIAL.

HOMŒOPATHY OR ECLECTICISM?—In our June issue we said: "We have endeavored to set plainly before the profession the fact that there are two distinct parties in the so-called homœopathic school. The one representing eclectic methods and practice; the other, the principles and practice of Hahnemann. The time has now come when all practitioners must choose which party they will aid and assist. Will you retrograde or advance; be an eclectic or an homœopathist?" Each meeting of the American Institute emphasizes these questions.

Guided by a clique, the Institute *retreats* year by year from the advanced position in which it was placed by its *homœopathic* founders in 1844. Eclecticism now rules its counsels: its debates and transactions show little of Homœopathy. Some years ago its "code" was remodeled; eclecticism was allowed, and now a candidate for membership is required to know *nothing* of Homœopathy.

At its recent meeting a resolution, somewhat as follows, was introduced and passed:

*Resolved*, That it is the sense of the American Institute that no physician can properly sustain the responsibilities or fulfill all the duties of his professional relations, unless he enjoys absolute freedom of medical opinion* and unrestricted liberty of professional action as provided for in the code of ethics of this Institute.

If "the code of ethics" provides for all this freedom, why was this resolution introduced?

* As an example of this freedom of opinion and action, the President of this noble Institute proposed to limit the dose, BY RESOLUTION, to the 10th potency!! You may be free, but must not go above the 10th potency!!!

The truth of this resolution is so self-evident that one is surprised that any one should consider its affirmation necessary. In this country, at least, all physicians enjoy unlimited freedom to think and to act as they please. Medical opinion is absolutely and entirely free: professional action is only restricted by the *coroner.* And as a physician uses this freedom, so is he classed as an eclectic or as an homœopathist. His actions make him one or the other; and resolutions do not affect this decision.

This resolution, coming from a body of professed homœopathists, simply means that they have found Homœopathy insufficient for all the needs of their patients. As this practice has for years been proclaimed all-sufficient, for all practical duties, this resolution is a distinct *back-down,* a disordered *retreat!* What now is the cause of this retreat? Have those who approve of this *back-down* fully tested the law of the Similars and found it wanting? If so, will they kindly inform the benighted believers in that law, under what conditions they find it a success and under what a failure? Illustrating and enforcing this by clinical cases. And what do they propose as assistants and substitutes for this law? They surely cannot propose to tear down, without rebuilding on surer foundation?

This is no occasion for bombastic resolutions or glittering generalities. Let us have *facts.* Any one who knows of methods for curing disease, more successful than those in vogue, and withholds them, justly looses professional caste and forfeits respect.

Unless the new methods are more scientific and more successful they cannot be considered an improvement or an advance.

The most successful men in our school have found their best results came from a strict adherence to the law of the Similars.

---

An old and honored member of the Institute writes us as follows: "I would emphasize the fact that these people who are everlastingly chattering about 'liberty of opinion' are, of all men, the most illiberal. They will not allow to any man the opinion that Homœopathy is based on God's law, and that God's law is mandatory, not permissive; that healers are under obligations to obey this law; that these drivelers are perfectly willing you and I shall believe anything in the world except God's law as given us by Hahnemann, and practice anything, with any means, except whatever is characteristic and true homœopathic philosophy and practice, their utmost stretch of liberality not being able or willing to include this in their toleration. And if any one is so weak or unwise as to believe and practice as Hahnemann taught, together with the growth this has received from the latest and best practical experiences, he is not to suffer a word of this to escape him—certainly not in the American Institute. If he does this, he will soon be made to know this Institute has no sympathy with him, his utterances or his practice.

They can be abundantly liberal as to any man's belief or practice of false theories, but as to any belief in God's truth or practice founded on it, they have no tolerance for it. No, never. If a man talks true Homœopathy to them he is soon made to know he has spoken in the wrong place, and where his truth and practice have long since ceased to have a place.

How much is this kind of liberality worth to any man or cause? If it has any meaning it is this—that in the judgment of these wiseacres, a lie is just as good as God's truth or better, and in this resolving body it seems to have a not very assuring preference."

